# Feature Selection and Pedestrian Detection Based on Sparse Representation

**DOI:** 10.1371/journal.pone.0134242

**Published:** 2015-08-21

**Authors:** Shihong Yao, Tao Wang, Weiming Shen, Shaoming Pan, Yanwen Chong, Fei Ding

**Affiliations:** 1 State Key Laboratory for Information Engineering in Surveying, Mapping and Remote Sensing, Wuhan University, Wuhan, China; 2 Information Processing and Communication Technology Lab, Shanghai Institute of Satellite Engineering, Shanghai, China; Leibniz Institute for Age Research, GERMANY

## Abstract

Pedestrian detection have been currently devoted to the extraction of effective pedestrian features, which has become one of the obstacles in pedestrian detection application according to the variety of pedestrian features and their large dimension. Based on the theoretical analysis of six frequently-used features, SIFT, SURF, Haar, HOG, LBP and LSS, and their comparison with experimental results, this paper screens out the sparse feature subsets via sparse representation to investigate whether the sparse subsets have the same description abilities and the most stable features. When any two of the six features are fused, the fusion feature is sparsely represented to obtain its important components. Sparse subsets of the fusion features can be rapidly generated by avoiding calculation of the corresponding index of dimension numbers of these feature descriptors; thus, the calculation speed of the feature dimension reduction is improved and the pedestrian detection time is reduced. Experimental results show that sparse feature subsets are capable of keeping the important components of these six feature descriptors. The sparse features of HOG and LSS possess the same description ability and consume less time compared with their full features. The ratios of the sparse feature subsets of HOG and LSS to their full sets are the highest among the six, and thus these two features can be used to best describe the characteristics of the pedestrian and the sparse feature subsets of the combination of HOG-LSS show better distinguishing ability and parsimony.

## Introduction

With the development of intelligent cities, computers are involved in the field of intelligent monitoring and intelligent transportation control to conduct pedestrian detection from a large number of images. Pedestrian detection, as an integral part of intelligent monitoring and intelligent transportation, is a challenging task because of its high standards in both detection speed and reliability in intelligent surveillance, the Advanced Driver Assistance Systems (ADASs) and the Intelligent Transportation System (ITS) which are components of a smart city [1].

Computers, along with the development of a smart city, are expected to replace the human brain and perform equivalent human visual functions, such as information gathering, processing and interpreting, and mapping relationships between images and image descriptors. This study focuses on how to extract object features from images. The fact that the object features differ from one another enables us to extract pedestrians from the complex environmental backgrounds to realize the pedestrian detection.

Much ongoing studies have focused how to extract the pedestrian features effectively. Scale Invariant Feature Transform (SIFT) [2], which was initiated by Lowe D G et al., makes it possible to address applications that require the rigid deformation and perspective deformation of the images. However, the development of this approach is obstructed by the high level of computation complexity. However, this limitation can be compensated for Speeded-Up Robust Features (SURF) [3], which employs integral image and box filter to improve and optimize SIFT features to reduce the computational cost. A simple rectangular feature that is similar to Haar wavelet was proposed by Viola P et al. [4]. The computational procedure of Haar feature might be easily affected by a complex background because of its simplicity. Being the focus of investigating pedestrian for a long time, Histogram of Oriented Gradient (HOG) [5] inherited the advantages of SIFT features and is robust for changes in clothing, colors, human body figure and height. Because the rectangle detection window could not handle rotational transformation, the pedestrian must be in an upright position. In response, Kittipanya-ngam P et al. [6] suggested a square-shaped detection window which could contain more variations of pedestrians. Local Binary Pattern (LBP), which was originally utilized for text classification, was not suitable for human detection and recognition because of its high complexity and lack of semantic consistency. To overcome these shortcomings, Mu Y et al. [7] proposed two variants of LBP, Semantic-LBP and Fourier-LBP, for human detection and recognition. Local Self-Similarity (LSS) feature was proposed by Shechtman E. et al. [8] based on the texture features of images to densely calculate local self-similarity descriptors. Liu J et al. [9] proposed two new texture features called Local Self-Similarities (LSS, C) and Fast Local Self-Similarities (FLSS, C), which were based on Cartesian location grid. These features could achieve more robust geometric translations invariance with less computing time and higher precision.

There is a consensus that no single feature has a perfect performance in pedestrian detection because every feature has its own limitations. Feature fusion has received considerable attention from researchers in pedestrian detection. Wang X et al. [10] combined tri-linear interpolated HOG with LBP to make a new feature set that is capable of handling partial occlusion. A combined strategy was proposed by Yuan Xin et al. [11] that was based on Haar and HOG features. These combined strategies can greatly accelerate detection speed and maintain a high accuracy for the HOG classifier. Based on the above-mentioned features, Walk S et al. [12] went further by incorporating the local color self-similarity and motion features, while Wu B et al. [13] combined HOG, edgelet and covariance feature to make a new feature.

Owing to the variety of features, feature selection in predictive modeling has received considerable attention in statistics and machine learning selection [14]. Feature selection can be broadly classified into three categories: wrapper, filter, and embedded methods. The wrapper methods perform a heuristic search in the space of all possible feature subsets, but the exponential number of possible subsets makes this method computationally expensive in general [15]. Filter methods which are independent of any classifiers, apply evaluation measures to select features and then build a classifier using the selected features [16–17]. Because of their simplicity and fast computational performance, many filter methods have been proposed for solving the feature selection problem [18–20]. The embedded methods, using classification or regression as an optimization with specified loss and penalty functions [21–23], attempt to simultaneously maximize the classification performance and minimize the number of features used [24]. These methods are more efficient than wrapper methods because a filter algorithm is built with a classifier to guide the feature selection procedure.

As discussed in the above researches, the following conclusions were obtained: (1) pedestrian features are the key factors in pedestrian detection; (2) the description ability and feature dimension of those features are vastly different; (3) the overall dimensions of the present features are relatively high and increase the detection time in a real-time human detection system. The commonly used feature descriptors carry all of the pedestrian information, but not all of them are useful. Our research focused on how to eliminate useless information and improve the speed of pedestrian detection.

On the other hand, sparse representation is an extremely effective tool for the applications such as image compression and denoising. These applications appear to be different but have a common goal of simplifying the signal representation.

Labusch K et al found [25] that their innovative method, based on sparse representation of local image patches, was superior to Gabor wavelets’ on the MNIST set recognition. This result showed that the method of sparse coding theory can be widely applied in pattern recognition. A robust method for face recognition via sparse representation was proposed by John Wright and Yi Ma [26], which was based on compressive sensing. This method aimed to explore the sparsest solution vector from the linear representations, whereby the optimal one for the testing sample is achieved by using the fewest training samples. Liu J et al. [27] extracted face features from the perspective of sparse theory in the same way. In Liu’s method, the algorithm constructs an objective function that projects the original data into a subspace with a maximum sub-manifold distance and minimum manifold locality. This method presented higher accuracy for face recognition and identification than the method in Qiao L et al. [28]. Instead of random sampling from the entire image, overcomplete dictionaries from random block sampling near facial salient points was designed and a new sparse formulation was created by Haiying Xia et al. [29] for recognizing facial expressions.

Based on the previous studies, we apply sparse theory to pedestrian feature detection and adopt a supervised key-feature subset selection method in our research. The full features are the entire original descriptors. For example, the full features of HOG with the 1575-dimensional vector are constituted by the sub-HOG with 175 blocks. The feature subsets with discrimination and parsimony are selected from the full features and thus provide a reliable theoretical basis for selecting the optimal features. Thus a sparse representation can be adopted to filter the features, eliminate the redundant features, reduce the feature dimensions, and further shorten the pedestrian detection time without impacting the detection performance.

In this paper, six of the most frequently-used features, SIFT, SURF, Haar, HOG, LBP, and LSS, are analyzed and compared. Then, a supervised key-feature subset selection method is used to select the most distinctive feature from the analyzed features. The Support Vector Machine (SVM) [30] and Adaboost [31] are selected as classifiers in that they are the most popular classifiers for their good performance and efficiency. We performed feature selection experiments starting from the combination of all possible feature types in [32]. From this research, we observed better combinations of features at the expense of the detection time because feature fusion can make the feature dimension increase. In view of this situation, we add sparse representation theory in this paper and attempt to reduce the feature dimension and detection time without affecting the feature description ability. Compared to [32], our selected feature descriptors of interest are chosen by multiple iterations and update and have more robustness for pedestrian description. Sparse features can be rapidly generated by avoiding calculations of the corresponding index of the dimension number of these feature descriptors; thus, the calculation speed for the feature dimension reduction is improved and the pedestrian detection time is reduced.

The pedestrian detectors are learned from the selected features using SVM and Adaboost in the experiments described in Section 4 and the selected feature subsets that own the same distinguishing ability as the original feature when different classifiers are identified.

The remainder of this paper is organized as follows. Section 2 introduces and analyzes the most frequently used features, SIFT, SURF, Haar, HOG, LBP, and LSS. Sparse theory and how to select the sparse feature subsets of the six features are described in Section 3. In Section 4, we verify the proposed method by performing extensive experiments on the INRIA dataset and Daimler dataset and compare the proposed method with other state-of-the-art pedestrian detection techniques. The capabilities of the proposed method for filtering and evaluating pedestrian features are also investigated.

## Image Feature Analyses

Six selected features, SIFT, SURF, Haar, HOG, LBP, and LSS are extracted from a typical image and are analyzed in detail below.

### 2.1 SIFT Feature

SIFT feature is a local feature descriptor that uses a difference-of-Gaussian function to identify potential interest points which are invariant to scale and orientation [2]. Based on the measures of their stabilities and contrasts, potential interest points are selected and transformed relative to the assigned orientation, scale, and location of each feature.

Furthermore, the local image gradients are measured at the selected scale in the region around each keypoint, and extreme points are sought from the adjacent three layers in the same octave.

### 2.2 SURF Feature

SURF feature, deduced from SIFT feature, approximates or even outperforms SIFT feature on repeatability, distinctiveness and robustness, and thus can be computed and compared much faster [3]. When SURF features construct a scale-space, the scale changes are achieved by altering the size of the box filter instead of altering the image size. The integral image is adopted in computing features to approximately simplify the filtering between the image and Gaussian second-order differential. Hence, it drastically reduces the number of operations for the simple box convolutions and is independent of the chosen scale. Only three additions and four memory accesses are needed to calculate the sum of the intensities in a rectangular region of any size.

### 2.3 Haar Feature

**Fig 1:**
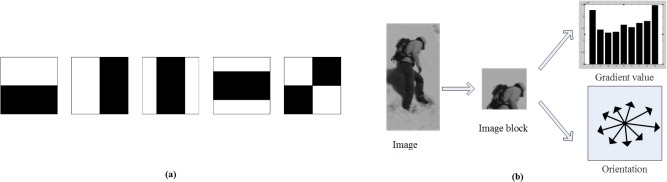
(a) Haar templates. (b) Gradient and orientation of HOG.

The value of Haar feature equals to the difference between the sum of the pixels in the white rectangles and those in the grey rectangles [4]. The template library of Haar feature includes the edge template, linear template, center template, and diagonal template, etc. The feature template can be set arbitrarily to any size in a sub-window. As shown in Fig 1(A), five Haar templates are selected and each image block has 5-dimensional Haar features. Once the forms of the templates are identified, the number of features can be determined by the size of the training sample images and the rectangle templates.

### 2.4 HOG Feature

HOG features are similar to Histograms of Edge Orientation features [33] and SIFT features. However, HOG descriptors are computed on a dense grid of uniformly spaced cells and overlap local contrast normalizations to improve performance [5]. As shown in Fig 1(B), a gradient histogram with 9 orientation bins is computed for each block, and the 9-dimensional HOG features are used in the experiments described in Section 4.

### 2.5 LBP Feature

LBP feature is updated as a general texture description operator for measuring and extracting local texture information from images [6]. The most influential advantages of LBP are its invariance to monotonic gray-scale changes, low computational complexity and convenient multi-scale extension. At each pixel, LBP can be defined as an ordered set of binary comparisons of pixel intensities between the center pixel and its eight surrounding pixels. LBP operators have three patterns: uniform pattern, rotation invariant pattern, and rotation invariant uniform pattern.

### 2.6 LSS Feature

**Fig 2:**
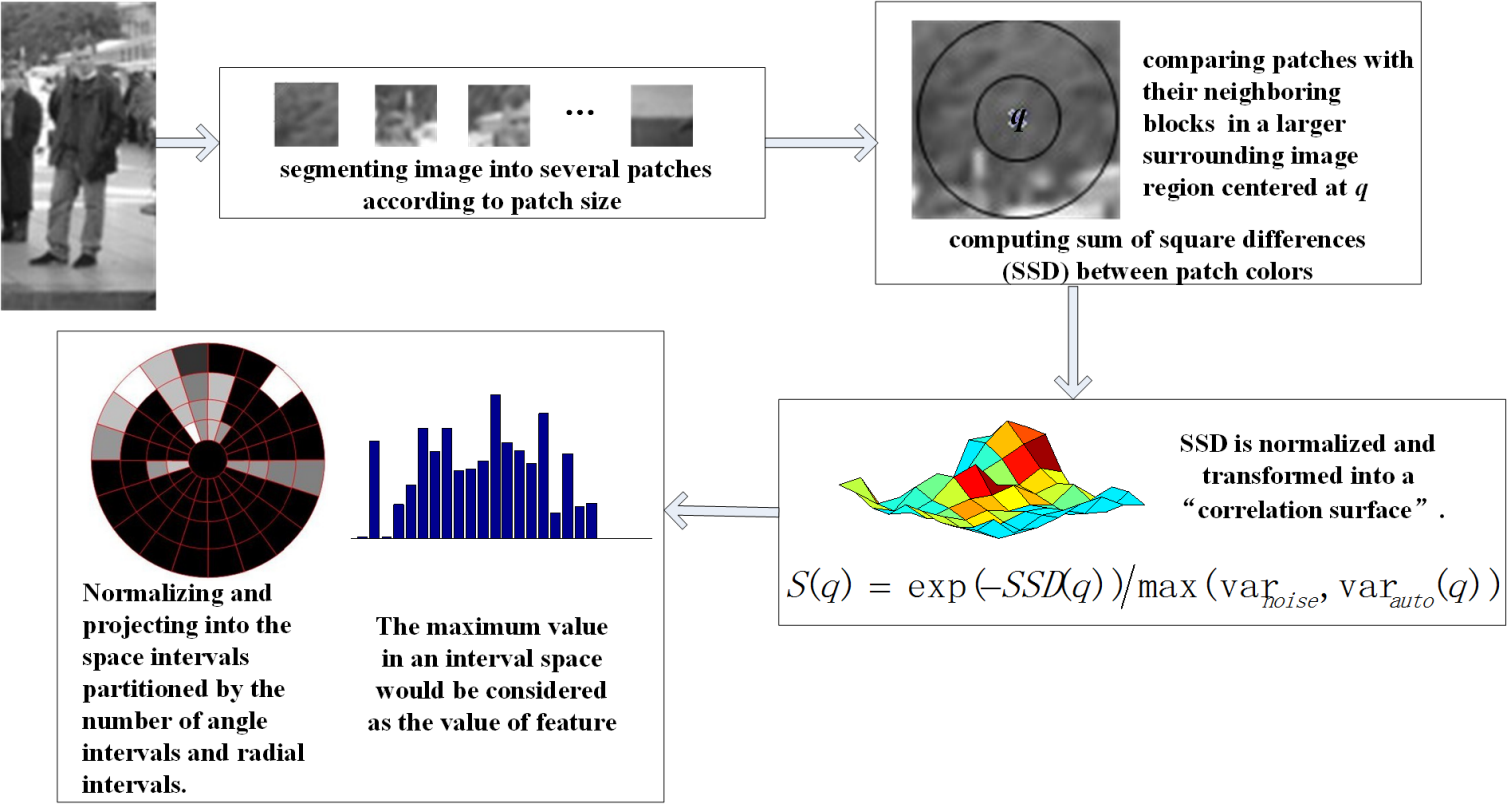
The process of LSS descriptor construction.

LSS features proposed by Eli Shechtman and Michal Irani are used for video and image matching [8]. As shown in Fig 2, the selected image block is compared with its neighboring blocks within a certain radius in a image region, in terms of the pixel value, the resulting SSD (Sum of Square Differences) is normalized and then projected into the space intervals that are partitioned by the number of angle intervals and radial intervals. The maximum value in an interval space is considered as the value of the feature. Here, var_noise_ is a constant that corresponds to acceptable photometric variations (in color, illumination or noise), and var_*auto(q)*_ takes into accounts for the patch contrast and its pattern structure, Thus, sharp edges are more tolerant to pattern variations than smooth patches.

### 2.7 Comparative Analysis of Feature Descriptors

Different feature descriptors have different description abilities. It is highly recommended that the six features should be analyzed based on their own characteristics. Based on the theoretical analysis of the six features, an optimal descriptor for pedestrian representation is discovered. As shown in Table 1, with the exception of HOG, the other five descriptors require the difference operators, which are obtained by subtraction between the image blocks pixels. SIFT, SURF and HOG descriptors are built based on gradient values, and they must convolve with filters to achieve a pyramid scale space. Besides Haar descriptor, the other five descriptors require a projection of the results or a histogram calculation. SIFT, SURF and HOG descriptors calculate histograms by using the angle interval of the pixels, while the LBP and LSS descriptors must project the results into their own templates.

**Table 1:** Analysis of six feature descriptors.

	descriptors					
Operator	SIFT	SURF	Haar	HOG	LBP	LSS
Difference	√	√	√		√	√
Gradient	√	√		√		
Convolution	√	√		√		
Projection(Histogram)	√	√		√	√	√

SURF and SIFT contain all four of the operators and possess comprehensive image information, but they are highly complex. HOG descriptors contain three operators, except for the difference operator. When HOG is combined with Haar, LBP and LSS descriptors respectively, the new combined features include all four of the operators. It is believed that human appearance can be better captured if the edge, local shape information and texture information are combined.

Haar features are called rectangular features. The sums of the pixels in the black rectangles and the white rectangles are calculated separately, and then the sums are subtracted. When the smooth patterns correspond to the different background of an image, the Haar features are almost equal to zero and have no discrimination. Thus, Haar descriptors are applied only in the images that have obvious edge changes. HOG is a descriptor that represents the local object appearance. HOG performs poorly while LBP and LSS are complementary when the background is cluttered with noisy edges [34]. LBP descriptors are obtained by comparing pixels and LSS descriptors, which are the comparison among image block. In terms of the difference operators in LBP and LSS descriptors, a pixel-based comparison is too fine to exactly describe the texture characteristics of an image. LSS descriptors are measured locally (within a surrounding image region). Consequently, the fusion feature of HOG and LSS descriptors contains the local information and entire information of the images. Thus, our study focuses on combining HOG with LSS descriptors and eliminating redundant information of the combined features.

### Sparse Feature Subset Extraction

Compressed sensing (CS) refers to as a random undersampling technique that breaks through the constraints on the sampling frequency in Nyquist sampling theory and acquires the entire signal information with fewer sparse sampling values [35]. Sparse representation theory is derived from the compressed sensing and is built on over-complete dictionary, which is an over-complete redundancy function. The element in the over-complete dictionary is called an atom. In this paper, in contrast with the conventional dictionary construction, pedestrian features are taken as the basic elements of the dictionary and the pedestrian is then represented by a linear combination of atoms. A small number of atoms (column vector) for representing pedestrians, denoted as *y*, are selected from the over-complete dictionary, denoted as *A*, so that the solution vector, denoted as *x*, can be the sparsest.

Given a sufficient number of training samples and the feature sets of the samples, *A* = [*α*_1_,*α*_2_,*α*_3_,⋯*α*_*i*_,⋯*α*_*n*_], where *A* ∈ *R* ^*m*×*n*^, *y* ∈ *R* ^*m*^, m is the dimension of features and n is the number of samples, and any test sample will lie approximately in the linear span of the training samples:

$$y=\alpha_{1}x_{1}+\alpha_{2}x_{2}+\alpha_{3}x_{3}+\cdots +\alpha_{i}x_{i}+\cdots +\alpha_{n}x_{n}$$ (1)

where *x*_*i*_ ∈ *R*, *i* = 1,2,⋯,*n*, and *α*_*i*_ represent the *i* ^*th*^ sample which is a m-dimensional vector and *α*_*i*_ ∈ *R* ^*m*^. We define a coefficient vector as *X* = [*x*_1_,*x*_2_,⋯,*x*_*n*_]^*T*^, and the linear representation of *y* can be rewritten as follows:

y=AX (2)

Based on the redundancy of dictionary, the vector *α*_*i*_ is no longer linearly independent, thus the solution of *y*, i.e., *x*_*i*_, in the Eq (1) is not unique. The overcomplete sparse representation involves finding the sparsest decomposition coefficient from all of the solutions. Because the entries of the vector *X* encode the identity of the test sample *y*, *X* can be obtained by solving the linear system of equations above (2). The solution of the equation is related to the dimension of the matrix *A*. If *m* > *n*, then system of equations (2) is overdetermined and the unique and correct solution of *X* can be determined. However, *m* is always smaller than *n* in pedestrian detection applications, and the system of equations (2) is typically underdetermined. Thus, the solution of *X* is not unique. If the samples in dictionary *A* are sufficient, then the sparse solution of *y* can be obtained by using the L1-minimization method.

The sparsest representation therefore automatically discriminates among the various classes presented in the training set [36]. In a pedestrian detection system, there are only two classes, and the pedestrian features are apparently different from other object features. The sparsest representation of the pedestrian feature is naturally discriminative: among all of the subsets of the base vectors, the sparsest representation results from selecting the subset that most compactly expresses pedestrian and rejects all of the other possible but less compact representations. The most effective features whose indexes are nonzero are selected. The output labels are determined from the selected sparsest subsets from the same class. In our pedestrian training procedure, we denote the index of a sample by the variable *i*, the label of the *i* ^*th*^ sample by the variable *y*_*i*_, the label set of all of the samples by the variable *Y*, the sparse coefficient, which is an n-dimensional vector, by the variable *W*, the vector that describes the pedestrian feature of the *i* ^*th*^ sample by the variable *v*_*i*_, the pedestrian feature set of all of the samples by the variable *V*, and the number of feature dimensions by the variable *m*. In addition, we define the following symbols to refer to the data that leads up to time L; the matrix in which *k* rows are selected from *V* is denoted by the variable *V*_*L*_; the labels of the corresponding *k* entries of *Y* are denoted by the variable *Y*_*L*_; and the corresponding sparse solution is denoted by the variable *W*_*L*_. The positive number *k* is set in advance.

Then, the linear representation of *y*_*i*_ can be rewritten as

$$y_{i}=Wv_{i}$$ (3)

where *y*_*i*_ is no longer a pedestrian feature, *y*_*i*_ ∈ {−1,+1}, -1 represents a negative sample and +1 represents a positive sample, *v*_*i*_ ∈ *R* ^*m*^.

Hence, we have

Y=VW (4)

where *Y* = [*y*_1_,*y*_2_,*y*_3_,⋯,*y*_*n*_]^*T*^, *V* ∈ *R* ^*m*×*n*^ and the *i* ^*th*^ row is *v*_*i*_, The solution of the system of equations in (4) is *W*, which can be approximately recovered by solving the following equation:

$$\min\;{\left\|W\right\|}_{1}\;s.t.\;Y=VW$$ (5)

In the experiments, we randomly select *k* rows from *V* = [*v*_1_,*v*_2_,*v*_3_,⋯*v*_*i*_,⋯*v*_*n*_]^*T*^ *L* times to construct a matrix denoted by *V*_*L*_, where *V*_*L*_ ∈ *R* ^*k*×*m*^, and the value of L is determined by the final termination conditions. Then, the corresponding *k* entries of *Y* form a new column vector that is denoted as *Y*_*L*_, *Y*_*L*_ ∈ *R* ^*k*^. The equations in (5) can be rewritten as

$$\min\;{\left\|W_{L}\right\|}_{1}\;s.t.\;Y_{L}=V_{L}W_{L}$$ (6)

The solution of the system of equations in (6) is denoted by ${\hat{W}}_{L}$. If we let

$$W_{L}=\frac{1}{L}\sum_{i=1}^{L}{\hat{W}}_{L}$$ (7)

when *L* > 1, and ‖*W*_*L*_−*W*_*L*−1_‖_2_ < *δ*, where *δ* is a given small positive constant, the iterative computations are stopped and *W* = *W*_*L*_. We define

$$F=\left\{i|\left|w_{i}\right|>\varepsilon ,i=1,\cdots ,m,w_{i}\in W\right\}$$ (8)

where *ε* is a given positive constant and *F* is the index of the entries of our selected feature descriptors of interest in *V*.

At each iteration, new index entries of the selected feature descriptors of interest are added in *W*_*L*_, and the sparse coefficients in *W*_*L*_ are updated continuously with new index entries of the selected feature descriptors. This process makes the selected feature more robust for pedestrian description.

In the following section, the six feature descriptors are denoted as vectors and concatenated to a new feature vector. By solving for the sparse coefficient *W*, the pedestrian description ability of each of the feature descriptors is tested and evaluated based on the distribution of sparse coefficient, where the three parameters *k*, *δ* and *ε* are set in advance.

## Experiments and Analysis

This paper intends to study the performance of various feature descriptors, and experiments on publicly available datasets for pedestrian detection are presented in this section. Because the INRIA and Daimler datasets are more comprehensive and complex than older or more limited datasets, such as MIT, NICTA and CVC datasets, we adopt these two datasets as experimental data. The training and testing procedures are conducted on the INRIA dataset, whose resolution is 128×64, and the Daimler dataset, whose resolution is 96×48. Next, 2300 positive samples of frontal and other views are selected randomly from the INRIA dataset and Daimler dataset, respectively, and 5000 negative samples are taken randomly from the INRIA dataset. To be consistent with INRIA dataset, the images in Daimler are resized from 96×48 to 128×64. In addition, SVM and Adaboost are the most popular classifiers for pedestrian detection. In the feature evaluation, Gentle Adaboost with a weak classifier based on a decision-tree that contains two branches at most and an SVM with a gamma kernel function (c = 2, g = 0.0078125) are used as classifiers. To evaluate the description abilities of the six features, 10-fold cross-validations are adopted in the experiments according to four criteria, which are the miss rate (miss rate = 1-detection rate), the false positives per window (FPPW), the false positives per image (FPPI) and the detection time.

The detection performance is affected by the selection of the three parameter values. The parameters *k* and *ε* are determined by many simulation experiments to reduce the stochastic component of the proposed algorithm. We adopt half of the INRIA dataset and Daimler dataset samples and combine them for use as an experiment dataset in the parameter selection process. We first set *δ* = 0.01, which meets the iterative convergence requirement and we attempted to perform simulation experiments to obtain optimal value for *k* and *ε*. Table 2 shows that the mean detection rate of the six sparse features for different value of *k* and *ε*. As shown in Table 2, under the same *ε*, the mean detection rates of the six sparse features do not change in a obvious fashion, however the mean detection rates of the six sparse features varied obviously under the same value of *k*. The range of the parameter *k* decides the sparse coefficient *W*, and the entries of *F* are profoundly affected by the choice of parameter *ε* which is affected by the coefficient *W*. The bigger the *ε* is, the higher the feature dimension is. If a larger value of *ε* is taken, feature dimension reduction cannot be achieved, and irrelevant and redundant information can not be eliminated.

**Table 2:** The mean detection rate of the six sparse features on different *k* and *ε*.

	*k*					
*ε*	0.2*n*	0.3*n*	0.4*n*	0.5*n*	0.6*n*	0.7*n*
0.2 max(*w*_*i*_)	0.6308	0.6375	0.6412	0.6425	0.6549	0.6510
0.3 max(*w*_*i*_)	0.6804	0.6822	0.6901	0.6981	0.7025	0.7065
0.4 max(*w*_*i*_)	0.6902	0.7153	0.7211	0.7354	**0.7416**	0.7352
0.5 max(*w*_*i*_)	0.6894	0.7016	0.7042	0.7145	0.7213	0.7248

From the above simulation experiments, we set parameters *k* = 0.6*n* and *ε* = 0.4 max(*w*_*i*_), where *n* is the number of samples. The entries of *F* are sparse and their corresponding features are used as samples.

### 4.1 Sparse Feature Comparative Experiments on the INRIA dataset

**Fig 3:**
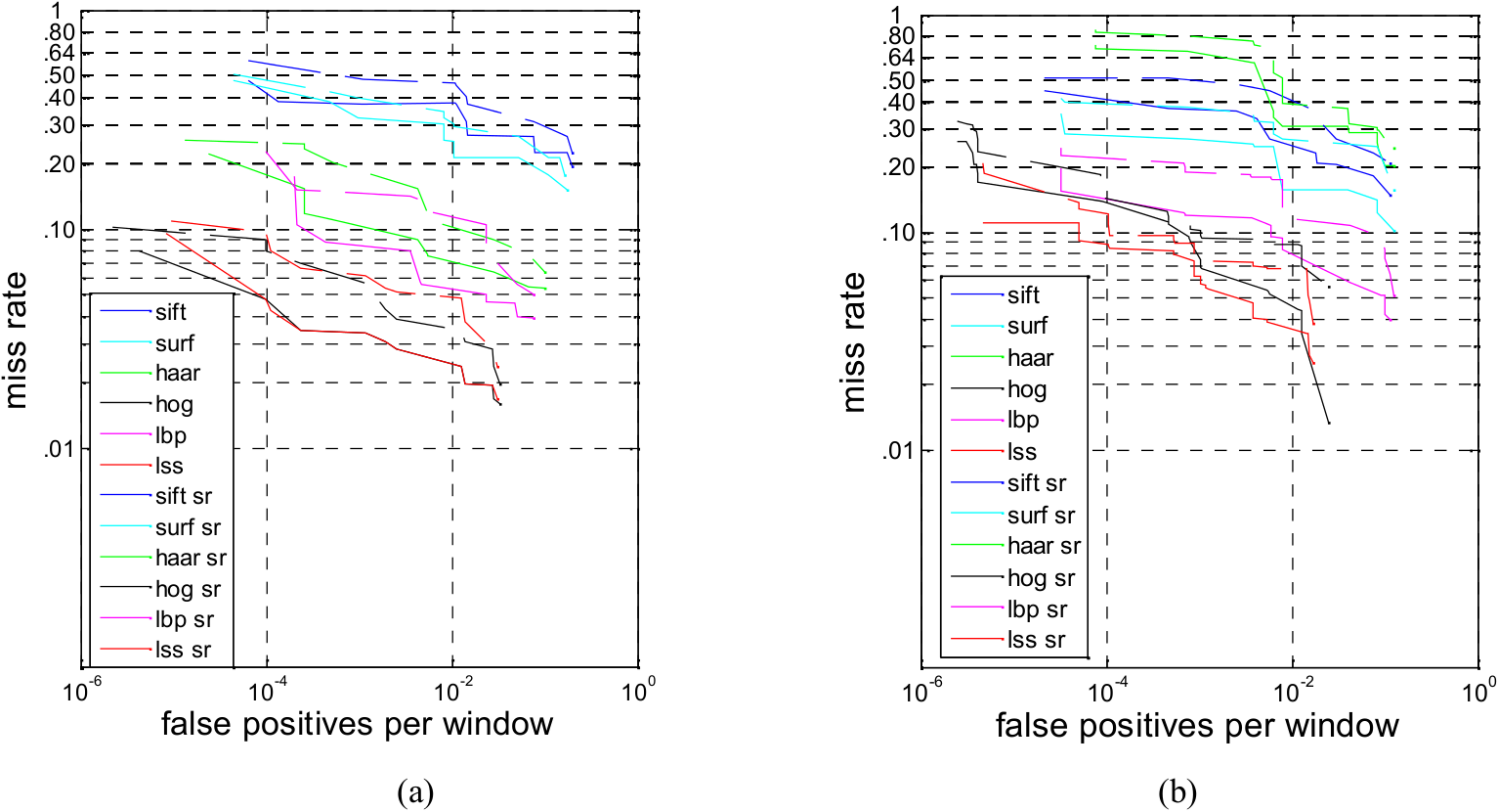
(a) The performance comparison among the sparse feature subsets and the full features on INRIA, with Adaboost is used as the classifier. (b) The performance comparison among the sparse feature subset and the full features on INRIA, with SVM is used as the classifier.

To verify the detection performance of the sparse feature subset on the pedestrian data, the INRIA dataset, which provides an extensive benchmark for testing various pedestrian detection methods, is first adopted with Adaboost as the classifier. We plot the miss rate versus the false positives per window (lower curves indicate better performance) and use the log-average miss rate as a common reference value for summarizing the performance. For the sake of performance comparison on the full features and sparse features, we add an experiment of the full features [32] in the following figures. Fig 3(A) shows the performance comparison among the sparse feature subsets and the full features on INRIA, and Adaboost is used as the classifier. The curve called ‘sift’ displays the performance of the full feature of SIFT, the curve called ‘sift sr’ displays the performance of the sparse feature of SIFT, and so on. In terms of the performance of a single feature descriptor, the HOG and LSS feature descriptors present the best performance in the single feature experiments, and LSS has slightly better performance than HOG. SURF and SIFT involve multi-scale features and have better performance in object matching. However, only selecting the keypoints features cannot meet the requirements of complex pedestrian detection. The construction of the Haar feature is simpler than the other features, and the descriptive ability of Haar is inferior to that of the HOG, LBP and LSS features. The performances of the six sparse features are lower than their full features, respectively, however the performances of LSS sparse feature subset and HOG sparse feature subset are still superior to the performances of the full features of SURF, SIFT, Haar, LBP and their corresponding sparse feature subsets. Under the same detection rate, the false positive rates of the sparse feature subsets are higher than the full feature’s rate, which reveals that the negatives are mistaken for samples with pedestrians because the number of features is significantly reduced.

Fig 3(B) shows the performance comparison among the sparse feature subsets and the full features on INRIA, with SVM as the classifier. The experimental results using the SVM classifier are roughly identical to the results when Adaboost is used as the classifier. Fig 3(B) shows that the performances of six sparse features are lower than their full features, especially the performance of the Haar sparse feature subset, which decreases sharply compared with the full feature of Haar. However, the HOG and LSS feature descriptors still present the best performances among the six features.

### 4.2 Sparse Feature Experiments on the Daimler dataset

**Fig 4:**
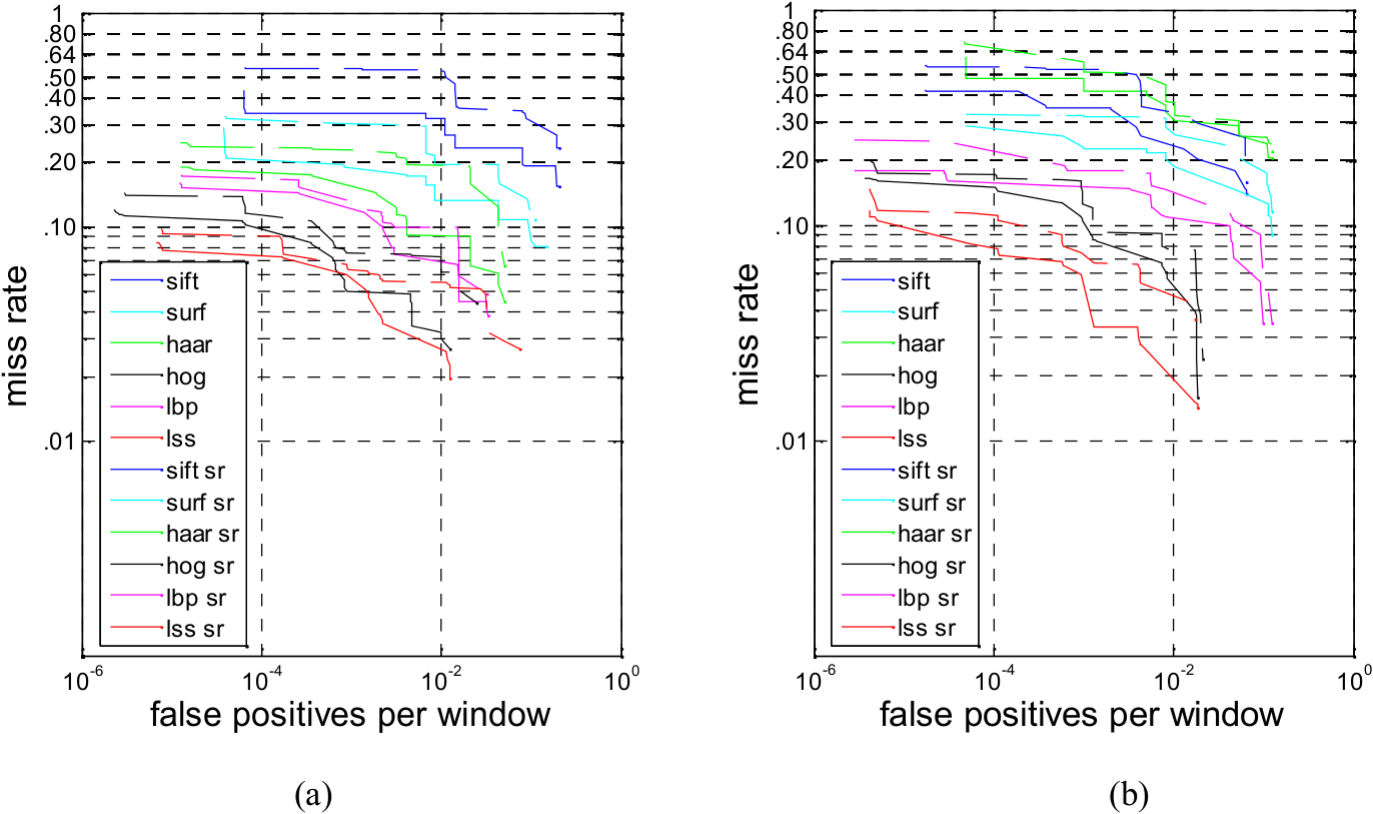
(a) The performance comparison among the sparse feature subset and the full features on Daimler, with Adaboost is used as the classifier. (b) The performance comparison among the sparse feature subset and the full features on Daimler, with SVM is used as the classifier.

To verify whether the sparse feature subsets have the same performance in the different datasets, we use the Daimler dataset in our experiments. Fig 4(A) shows the performance comparison of the sparse feature subsets and full features, and Adaboost is used as the classifier. As shown in Fig 4(A), the performance of the full features is better than that of the sparse feature subsets’. Taking 10^−2^ of FPPW as a reference line, compared with the full features, the detection rate of the Haar sparse feature subsets decreases by 11.65%, the detection rate of the SIFT and SURF sparse feature subsets decrease by approximately 15%, and the detection rate of the HOG, LBP and LSS sparse feature subsets decrease by approximately 3%. The detection rate of the LSS sparse feature subset is the highest among these six sparse feature subsets.

Fig 4(B) shows the tendency of the miss rate versus the false positives per window for sparse feature subsets and the full features, with SVM as the classifier. Here, 10^−2^ of FPPW is taken as a reference line, and HOG and LSS decrease the least.

Table 3 presents the feature dimension of the sparse feature subsets and the full features. Obviously, the feature dimension of the sparse feature subsets is decreased by almost 10 times.

**Table 3:** The feature dimension of the sparse feature subsets and the full features.

Feature	SIFT	SURF	Haar	HOG	LBP	LSS
INRIA dataset Dim(sparse)	134	258	126	187	112	251
Daimler dataset Dim(sparse)	133	166	72	244	134	218
Dim(full)	1408	2368	1420	1575	1450	1920

**Fig 5:**
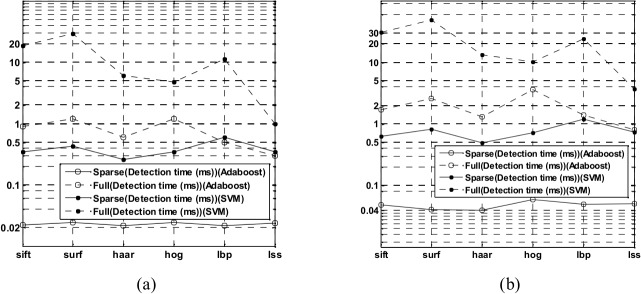
(a) The detection time of the sparse feature subsets and the full features on the INRIA dataset. (b) The detection time of the sparse feature subsets and full features on the Daimler dataset.

Pedestrian detection is under constant pressure to increase both its quality and speed. Such progress allows for new applications. A higher speed enables its inclusion into larger systems that can perform extensive subsequent processing. The detection time is a critical indicator for pedestrian detection. Fig 5 shows the detection time of the sparse feature subsets and the full features with different classifiers on the two datasets. When Adaboost is taken as the classifier, the detection time for the full features is approximately 10~15 times greater than that of the sparse feature subsets. When SVM is used as the classifier, the detection time of the full features is approximately 4~20 times greater than that of the sparse feature subsets. Regardless of which classifier is used, the detection time of the sparse feature subsets is far less than that of the full features.

In conclusion, the HOG and LSS feature descriptors have the strongest description ability and their sparse feature subsets retain the discrimination ability of the full features. Furthermore, their feature dimensions decrease greatly, and their detection speed is largely improved. Regardless of whether the analysis is on the INRIA dataset or Daimler dataset, the pedestrian description abilities of the sparse feature subsets are slightly weaker than those of the full features, but the detection time of the sparse feature subsets is significantly improved.

### 4.3 Experiments on the Sparse Fusion Feature subset

**Fig 6:**
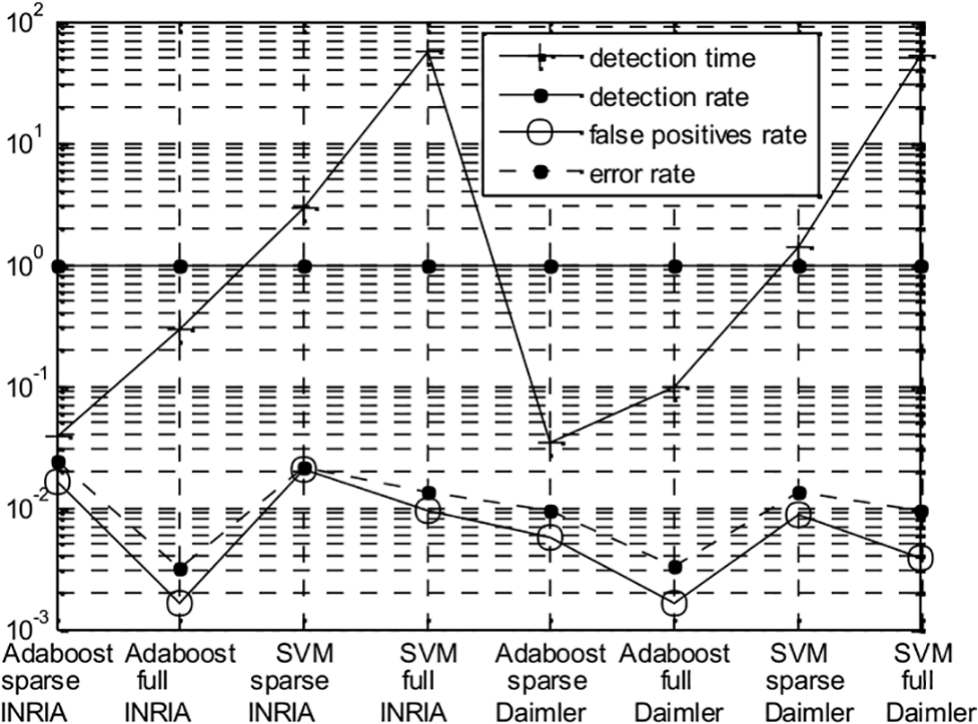
Performance indicators of the sparse fusion feature subset and the full feature on different classifiers.

To further study the feature selection ability of the sparse representation, these six features are fused into a new feature descriptor, and the sparse subset of this new feature descriptor is extracted. The SIFT, SURF, Haar, HOG, LBP, and LSS of the image *i* are given as *x*_*i*1_,*x*_*i*2_,⋯,*x*_*i*6_ respectively, and the full feature can be written as *x*_*i*_ = [*x*_*i*1_,*x*_*i*2_,⋯,*x*_*i*6_], which is a combination of the six features. The sparse fusion feature set $x_{i}^{\prime }=\left[x_{i1}{}^{\prime },x_{i2}{}^{\prime },\cdots ,x_{i6}{}^{\prime }\right]$ can be extracted with the coefficient vector *W* according to the computing process of section 3, where $x_{i}^{\prime }$ is the subset of *x*_*i*_. With the establishment of index relationships among the features, there is no need to solve Eq (6) in the process of features generation and pedestrian detection. The sparse feature subsets can be directly generated on the basis of the coefficient *W* in the subsequent procedure. Based on the experimental results on the INRIA and Daimler dataset, Fig 6 shows the four performance indicators for the sparse feature subset and the full features, which include the detection time, detection rate, false positives rate and error rate ($\text{Error}\;\text{rate}=\frac{\text{FalseNeg}\;+\;\text{FalsePos}}{\text{Positives}\;+\;\text{Negatives}}$) for different classifiers. We can find that the sparse fusion feature subset shows a better description ability regardless of whether the Adaboost or SVM classifiers is used.

Table 4 shows the composition and proportion of the sparse fusion feature on the INRIA and Daimler dataset. The total dimension of the fusion feature is 10141, and the sparse fusion feature subset is 967 after sparse screening; thus, the feature dimension is reduced by approximately 10 times. In Table 4, lines 1 and 3 represent the dimensions of each feature in the sparse fusion feature subset; lines 2 and 4 represent the ratio of the each feature in the sparse fusion feature subset; and line 5 represents the dimension of each full feature.

**Table 4:** The proportion of sparse fusion feature.

Feature		SIFT	SURF	Haar	HOG	LBP	LSS
INRIA dataset	Dim(sparse)	165	96	1	337	68	300
	Percent (%)	17.06	9.93	0.11	34.85	7.03	31.02
Daimler dataset	Dim(sparse)	140	179	8	294	86	260
	Percent (%)	14.47	18.51	0.84	30.4	8.89	26.89
Dim(original)		1408	2368	1420	1575	1450	1920

Table 4 reveals that the proportion of HOG and LSS is more than 30% in the sparse fusion feature. The sparse subset of the fusion feature is mostly extracted from HOG and LSS, and the proportion of Haar is only 0.11% or 0.84%. The proportion of SURF increases from 9.93% to 18.51% because the INRIA dataset contains images of high-resolution pedestrians that were collected mostly from holiday photos, while the Daimler dataset contains only grayscale images that were recorded with a moving camera in urban environments. SURF has more robustness for grayscale images.

According to the comprehensive analysis above, it can be concluded that the sparse fusion feature subset has a good pedestrian description ability, and that the proportion of a single feature in a fusion feature is nearly invariable in the two datasets for two classifiers. This finding proves that the sparse representation method has good feature selection ability and that the proportion of features can evaluate the description ability of various features components in pedestrian detection. The proportion of HOG and LSS are higher than the others in the full feature set, that which means that HOG and LSS have the strongest pedestrian description ability in the six features.

**Fig 7:**
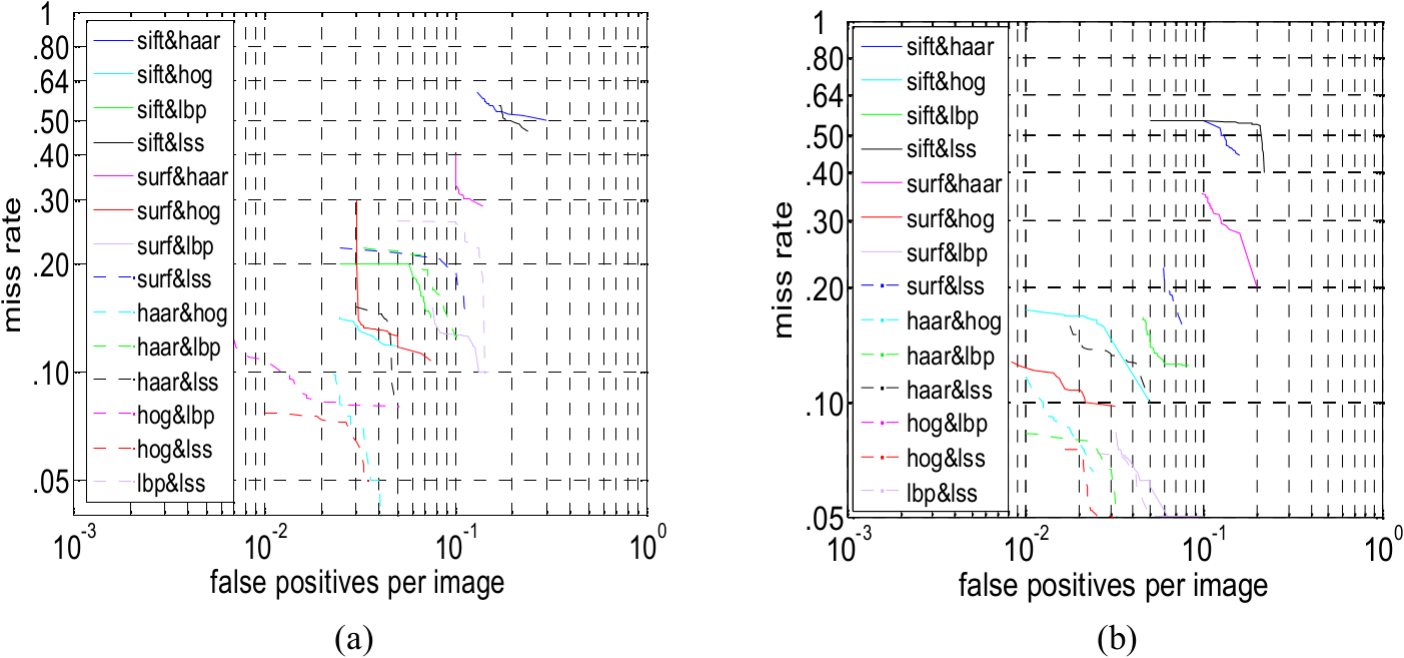
(a) Performance comparison between the sparse combined features (INRIA). (b) Performance comparison between the sparse combined features (Daimler).

Thus we combine HOG and LSS to generate a novel feature and calculate their sparse feature subset. Because the detection time on Adaboost is shorter, Adaboost is used as the classifier in the subsequent sparse combined feature experiments; in addition, the test images only contained only 1–4 persons. Any two of these six features are combined and form new combined features. In this paper, we study only the description abilities of these sparse combined features in the following experiments. Fig 7 shows the performance comparison between the sparse combined features on INRIA and Daimler, respectively. As shown in Fig 7(A) and 7(B), the detection performance of SIFT-Haar is the poorest, and HOG-LSS is the best on the INRIA and Daimler dataset.

Sparse features that are obtained by the feature selection method in Section 3 are clearly different from the full features. Sparse features have little redundancy and useless information. The detection time using sparse features is far less than that of the full features.

To further verify the robustness and stability of the pedestrian detection, we choose five typical and state-of-the-art pedestrian detection methods, Shapelet [37], HOGLbp [10], LatSVM-V2 [38], VJ [39] and HOG [5], for comparison with the HOG-LSS sparse feature with MutualCascade Adaboost [40]. MutualCascade improves the classic method of Cascade to remove irrelevant and redundant features. The mutual correlation coefficient is utilized as a criterion for determining whether a feature should be chosen or not. In this algorithm, a new updating mechanism for negative samples is utilized to ensure that the number of the negative samples at any level is the same as the original number during training. The updating mechanism is as follows: emptying the negative samples, we add not only the negative samples that are false positives at this stage but also negative samples from the optional negative samples library, which are false positives after being detected through all of the former stages.

**Fig 8:**
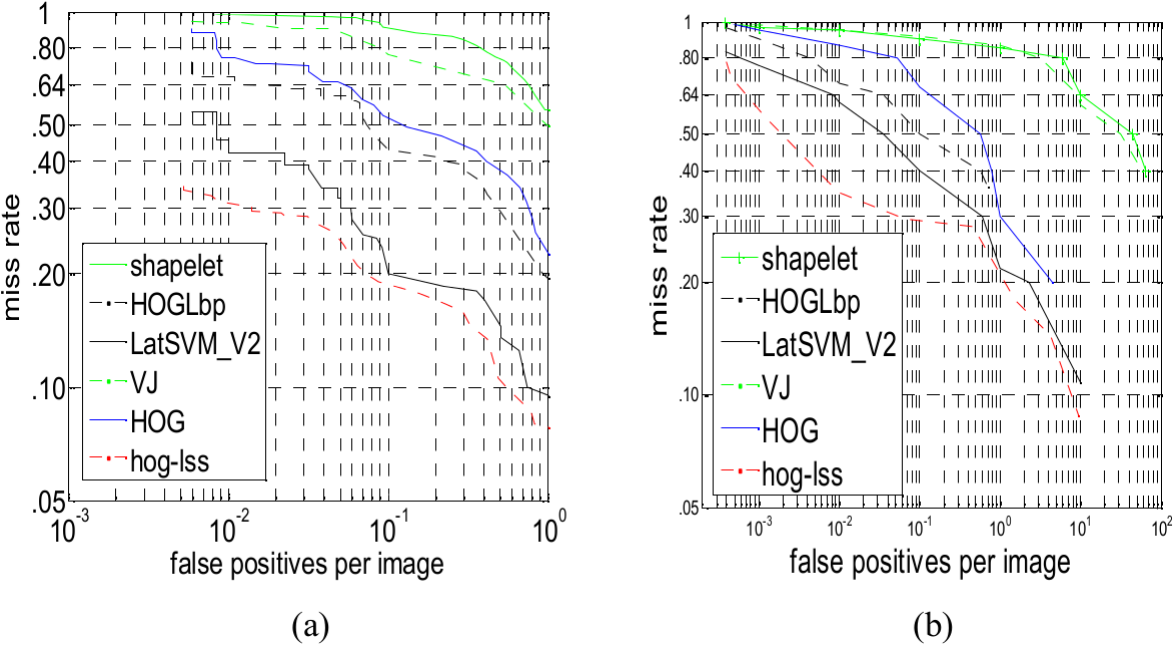
(a) Performance comparison between the HOG-LSS sparse feature and other methods on INRIA. (b) Performance comparison between the HOG-LSS sparse feature and other methods on Daimler.

Five different systems have been tested on two datasets, and the detection rates are available at a false positive rate of 10^−1^. Fig 8 shows the performance comparison between the HOG-LSS sparse feature and other methods on the INRIA dataset and Daimler dataset. Of the two datasets, the performance appears to be the best on INRIA, which contains high-resolution pedestrians, with the HOG-LSS sparse feature achieving a log-average miss rate of 17%-18% (see Fig 8(A)). The performance is also fairly high with a 20%-22% log-average miss rate attained by LatSVM-V2. HOGLbp is more challenging, with a log-average miss rate (loss rate) of approximately 43%. The log-average miss rate of HOG is 50%-52%. Shapelet and VJ are not ideal, with log-average miss rates of 77% and 89%-91%, respectively. As shown in Fig 8(B), LatSVM-V2 has the best performance in these five representative pedestrian detection methods, with a log-average miss rate of approximately 40%, and our method achieves log-average miss rates of 28%-29%. These prove that our method has distinct advantages compared with other methods on two datasets and that the HOG-LSS sparse features have parsimony and good pedestrian description ability.

**Fig 9:**
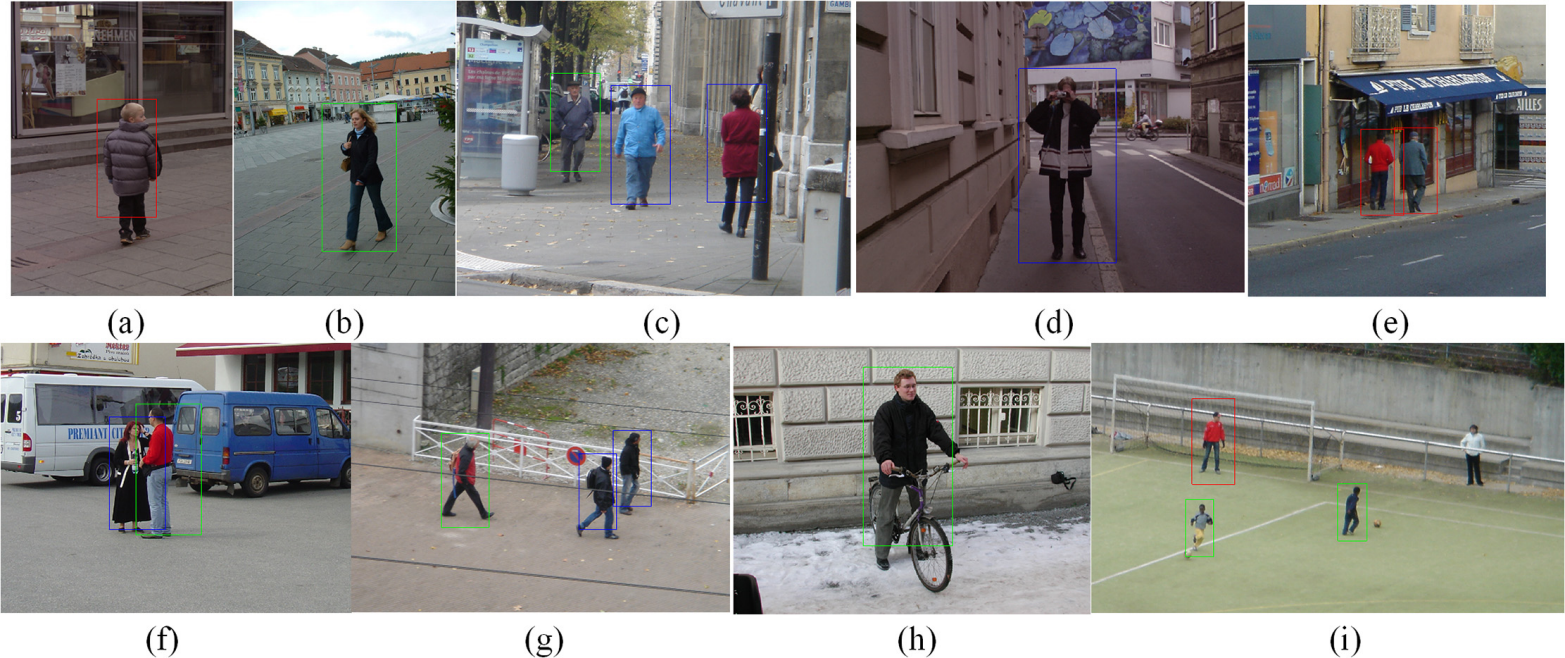
Sample detections on images that were densely scanned by the HOG-LSS sparse feature detectors.

Fig 9 presents several detection results on images that are densely scanned by the HOG-LSS sparse feature detectors. As shown in Fig 9, most of the pedestrians are correctly detected. The detection boxes with different colors in the images represent the detection windows with different scales. In Fig 9(C), a large part of the missing pedestrian is occluded by a signposts and the other missing pedestrian is a far-scale pedestrian with a height of at least 50 pixels and partial occlusions. In Fig 9(D), the missing pedestrian is the far-scale pedestrian with a height of at least 50 pixels. In Fig 9(I), the missing positive is divided into two parts by the stadium guardrail. The performance of the pedestrian detection is excellent in the near and medium scales but degrades seriously in the far scale. In this paper, we choose the detection at the medium scale as our research target, which meets the requirements of real systems.

## Conclusions

The variety and high dimensions of the pedestrian features are critical problems in pedestrian detection. In this paper, six frequently used features are analyzed in detail, and HOG-LSS would be considered as a new pedestrian detection feature that contains all of the image descriptive operators. We find that the sparse representation method has the ability to perform feature selection, which can remove redundant information and shorten the feature dimension, and a supervised key-feature subset selection method is used to select the most distinctive features from the analyzed features. Experimental results prove that sparse feature subsets can keep the important components of the six feature descriptors and that the HOG and LSS sparse features possess the equivalent description ability while consuming less computing time compared with the full feature. This paper solves the problem described in the literature [10] that there is lack of a theoretical basis for the feature combinations. The proportion of sparse feature subsets can evaluate the representation ability of feature components for pedestrians. HOG and LSS present the highest ratio in the full feature set, and as a result, these two features can best describe the characteristics of a pedestrian, and the sparse feature subsets of their combination show better discrimination and parsimony. Accordingly, the fusion feature HOG-LSS can be applied and popularized in practice and deserve further research and testing. A feature learning approach is attractive for its scalability and adaptability, which calls for future work on designing and learning much richer features.
